# Indoleamine 2,3-Dioxygenase 2 Immunohistochemical Expression in Resected Human Non-small Cell Lung Cancer: A Potential New Prognostic Tool

**DOI:** 10.3389/fimmu.2020.00839

**Published:** 2020-05-27

**Authors:** Martina Mandarano, Guido Bellezza, Maria Laura Belladonna, Jacopo Vannucci, Alessio Gili, Ivana Ferri, Chiara Lupi, Vienna Ludovini, Giulia Falabella, Giulio Metro, Giada Mondanelli, Rita Chiari, Lucio Cagini, Fabrizio Stracci, Fausto Roila, Francesco Puma, Claudia Volpi, Angelo Sidoni

**Affiliations:** ^1^Section of Anatomic Pathology and Histology, Department of Experimental Medicine, Medical School, University of Perugia, Perugia, Italy; ^2^Section of Pharmacology, Department of Experimental Medicine, University of Perugia, Perugia, Italy; ^3^Department of Thoracic Surgery, Medical School, University of Perugia, Perugia, Italy; ^4^Section of Public Health, Department of Experimental Medicine, University of Perugia, Perugia, Italy; ^5^Umbria Cancer Registry, Perugia, Italy; ^6^Department of Medical Oncology, Santa Maria della Misericordia Hospital, Perugia, Italy; ^7^Medical Oncology, Ospedali Riuniti Padova sud, Padova, Italy

**Keywords:** indoleamine 2, 3-dioxygenase 2, non-small cell lung cancer, immunohistochemistry, biomarker, immunomodulator

## Abstract

Indoleamine 2,3-dioxygenase 2 (IDO2) is an analog of the tryptophan degrading and immunomodulating enzyme indoleamine 2,3-dioxygenase 1 (IDO1). Although the role of IDO1 is largely understood, the function of IDO2 is not yet well-elucidated. IDO2 overexpression was documented in some human tumors, but the linkage between IDO2 expression and cancer progression is still unclear, in particular in non-small cell lung cancer (NSCLC). Immunohistochemical expression and cellular localization of IDO2 was evaluated on 191 formalin-fixed and paraffin-embedded resected NSCLC. Correlations between IDO2 expression, clinical-pathological data, tumor-infiltrating lymphocytes (TILs), immunosuppressive tumor molecules (IDO1 and programmed cell death ligand-1 – PD-L1 –) and patients' prognosis were evaluated. IDO2 high expression is strictly related to high PD-L1 level among squamous cell carcinomas group (*p* = 0.012), to either intratumoral or mixed localization of TILs (*p* < 0.001) and to adenocarcinoma histotype (*p* < 0.001). Furthermore, a significant correlation between IDO2 high expression and poor non-small cell lung cancer prognosis was detected (*p* = 0.011). The current study reaches interesting knowledge about IDO2 in non-small cell lung cancer. The close relationship between IDO2 expression, PD-L1 increased levels, TILs localization and NSCLC poor prognosis, assumed IDO2 as a potential prognostic biomarker to be exploited for optimizing innovative combined therapies with immune checkpoint inhibitors.

## Introduction

Lung cancer is one of the major cause of cancer-related morbidity and mortality across the globe, and non–small cell lung cancer (NSCLC) represents the majority of lung malignancies (1). In recent years, the treatment of NSCLC has been partly improved by the introduction of immunotherapies and, in particular, employing the FDA approved immune checkpoint inhibitors (2). However, only about 20% of these patients can benefit from this therapy, resulting in the need for new biomarkers both to amplify the effect of immune checkpoint inhibitors and to identify new and more efficient therapeutic targets (3).

A large body of evidence indicates that tryptophan (Trp) metabolism is of paramount importance in cancer progression and for the increase of malignant properties of cancer cells (4–6). The immunoregulatory molecule indoleamine 2,3-dioxygenase 1 (IDO1)—which catalyzes the first, rate-limiting step of Trp degradation through the kynurenine (Kyn) pathway—is highly expressed in many types of human cancers (6, 7) and is generally associated with poor prognosis (8). Similarly, tryptophan 2,3-dioxygenase (TDO), which catalyzes the same reaction of IDO1, is expressed in a wide range of malignancies and has been shown to promote tumor progression and metastasis (9). Less is known about the third member of the Trp-degrading enzyme family, indoleamine 2,3-dioxygenase 2 (IDO2) (6). IDO1 and IDO2 are closely linked on chromosome 8 in humans, probably originating from an ancient gene duplication which occurred prior to the evolution of vertebrates (10, 11). Although characterized by a high level of sequence identity (11), IDO1 and IDO2 exhibit important functional differences, such as IDO2 being endowed with a very weak catalytic activity *in vitro* (12). Moreover, plasmatic levels of Trp and Kyn are similar in wild-type and *Ido2* ^−/−^ mice, suggesting that IDO2 is not as efficient as IDO1 or TDO in converting Trp to Kyn *in vivo* (13). In tumors, IDO2 seems to be less frequently overexpressed than IDO1. Human gastric, colorectal, and renal carcinomas constitutively express both IDO1 and IDO2 (6, 14), as well as brain tumors, such as gliomas and meningiomas (15), and pancreatic ductal adenocarcinomas, in which IDO2 appears to be overexpressed (16).

However, despite the evidence of IDO2 expression in several types of malignancies, there are a limited number of studies about it in human tissues and its supposed functional role in the development and/or progression of cancer is still to be corroborated, in particular in NSCLC (6).

Recent studies showed that IDO1 is commonly expressed by NSCLC (17, 18) while there is still no evidence about its paralogue IDO2.

Our purpose is to evaluate the level of IDO2 through its immunohistochemical expression in a series of resected NSCLCs, in order to assess its presence and localization in the tumor cells of this specific type of cancer. Moreover, we aim to unveil potential correlations between IDO2 expression, clinical-pathological parameters, immunosuppressive molecules of the tumor microenvironment and patients' prognosis, in order to outline IDO2 as both a potential new biomarker for better patient risk stratification and as a possible target for the pharmacological treatment of NSCLC.

## Materials and Methods

### Patient Selection

The study has been prepared according to ethical guidlines regarding the informed consent of the involved human participants (Number of Local Ethic Committee Decision: 2216/13 of CEAS Umbria).

Patients were recruited from the computer archive of the Institute of Anatomic Pathology and Histology, S. M. Misericordia Hospital, Perugia, Italy, involving all the NSCLC cases which underwent a surgical resection in the period from 2009 to 2015. Moreover, only the cases with both known clinical parameters (summarized in Table 1) and with a complete clinical follow-up until 31st December 2017 were considered. The cases in pathological stage IV, according to the 8th edition for cancer staging by the American Joint Committee on Cancer (AJCC), were not taken into account. Regarding the other stages of disease, we arranged the NSCLCs into two groups: a Stage I group, encompassing the stages from IA1 to IB, and a Stage II-III one, enclosing the stages from IIA to IIIB.

**Table 1:** Expression of IDO2, clinical-pathological parameters and other microenvironmental molecule associations.

**Parameter**	**IDO2 low**		**IDO2 high**			**Total**	
	***N***	***%***	***N***	***%***	***p***	***N***	***%***
	**31**	**16**	**160**	**84**		**191**	**100**
**GENDER**							
M	23	17	114	83	0.739	137	72
F	8	15	46	85		54	28
**AGE**							
<68 years	13	15	74	85	0.659	87	46
≥68 years	18	17	86	83		104	54
**SMOKING**							
Current smokers	13	17	64	83	0.910	77	40
Former smokers	16	16	82	84		98	51
Never smokers	2	12	14	88		16	9
**RELAPSE**							
Yes	12	16	61	84	0.951	73	38
No	19	16	99	84		118	62
**EXITUS**							
Yes	6	9	58	91	0.068	64	34
No	25	20	102	80		127	66
**STAGE**							
***Adc**^***a***^**stage***						***122***	***64***
I	6	8	68	92	0.964	74	61
II - III	4	8	44	92		48	39
***Sqcc**^***b***^**stage***						***69***	***36***
I	7	26	20	74	0.513	27	39
II - III	14	33	28	67		42	61
**HISTOTYPE**							
Adc^a^	10	8	112	92	** <0.001**	122	64
Sqcc*^*b*^*	21	30	48	70		69	36
***Adc***^a^ ***pattern***						***122***	***64***
Other than solid	6	6	89	94	0.155	95	78
Solid	4	15	23	85		27	22
**TILs DENSITY**							
***Adc**^***a***^*						***122***	***64***
Low	5	8	57	92	0.956	62	51
High	5	8	55	92		60	49
***Sqcc**^***b***^*						***69***	***36***
Low	12	34	23	66	0.480	35	51
High	9	26	25	74		34	49
**TILs LOCALIZATION**							
***Adc**^***a***^*						***122***	***64***
Intratumoral	4	6	61	94	** <0.001**	65	53
Peritumoral	5	71	2	29		7	6
Mixed	4	9	42	91		46	38
Absent	0	0	4	100		4	3
***Sqcc**^***b***^*						***69***	***36***
Intratumoral	8	33	16	67	0.905	24	35
Peritumoral	2	29	5	71		7	10
Mixed	11	30	26	70		37	54
Absent	0	0	1	100		1	1
**IDO1**							
***Adc**^***a***^*						***122***	***64***
Low	4	7	52	93	0.695	56	46
High	6	9	60	91		66	54
***Sqcc**^***b***^*						***69***	***36***
Low	10	32	21	68	0.766	31	45
High	11	29	27	71		38	55
**PD-L1**							
***Adc**^***a***^*						***122***	***64***
Low	6	6	96	94	**0.035**	102	84
High	4	20	16	80		20	16
***Sqcc**^***b***^*						***69***	***36***
Low	19	40	29	60	**0.012**	48	70
High	2	10	19	90		21	30

a ***Adc:** adenocarcinoma*.

b ***Sqcc:** squamous cell carcinoma*.

### Histology and Immunohistochemistry

Surgical specimens were formalin-fixed (10% buffered formalin) and paraffin-embedded (FFPE). Sections of 4 μm were taken and placed on slides with a permanent positive charged surface, both to obtain the Hematoxylin and Eosin (H&E) stain and the Immunohistochemical (IHC) stains. The H&E stain was carried out using a Leica ST5020 Multistainer (Leica Microsystems), employing the kit ST Infinity H&E Staining System (Leica Biosystems). All the IHC stains (peroxidase immunoenzymatic reaction with development in diaminobenzinidine) were obtained by employing the BOND-III fully automated immunohistochemistry stainer (Leica Biosystems). In particular, IDO2 immunohistochemical slides were carried out using a heat-induced antigen retrieval with the ready to use Bond™ Epitope Retrieval Solution 1 (Leica Biosystems, Catalog No: AR9961) for 20 min, primary antibody incubation for 15 min (IDO2, Thermofisher Scientific, Cat# PA5-71696, RRID: AB_2717550, dilution 1:500) and the ready to use Bond™ Polymer Refine Detection System (Leica Biosystems, Catalog No: DS9800). Proper positive and negative controls were included.

Histological subtype was assigned based on H&E slides, according to 2015 World Health Organization (WHO) classification for lung tumors. Moreover, in line with the immunohistochemical expression both of TTF-1 (Agilent, Cat#M357501-2, RRID: AB_2801260; dilution 1:100; BOND-III fully automated immunohistochemistry stainer, Leica Biosystems) and p40 (ScyTek Laboratories, Cat#A00112-C, RRID: AB_2800554, dilution 1:50; BOND-III fully automated immunohistochemistry stainer, Leica Biosystems) poorly differentiated NSCLCs were classified as adenocarcinomas or as squamous cell carcinomas.

The H&E slides were also employed to determine both the localization of tumor-infiltrating lymphocytes (TILs) (absent; intratumoral= among tumor cells; peritumoral= at the interface between the neoplasia and healthy lung parenchyma; mixed= mixture of the last two localizations) and the density of TILs, according to the percentage of lymphocytes observed in a given localization (Low < 20%; High ≥ 20%) (19).

The immunohistochemical stains for IDO2 were evaluated on neoplastic cells and were interpreted, as previously reported (19), using an H Score resulting from the sum of the intensity of the stain (evaluated as 0: absent; 1+: mild; 2+: moderate; 3+: intense) and the percentage of the tumor cells labeled (0: 0%; 1: 1–25%; 2: 26–50%; 3: 51–75%; 4: 76–100%). Thereafter, two groups of staining were obtained: a low expression one—scores from 0 to 2—and a high expression one—scores from 3 to 7.

In addition, the results concerning the expression of both indoleamine 2,3-dioxygenase (IDO1) [courtesy of professor Benoit J Van den Eynde, Ludwing Institute for Cancer Research, clone 4.16H1 (7); dilution 1:1000; BOND-III fully automated immunohistochemistry stainer, Leica Biosystems] and programmed cell death Ligand-1 (PD-L1) (Cell Signaling Technology, Cat# 13684S, RRID: AB_2687655, dilution 1:200; BOND-III fully automated immunohistochemistry stainer, Leica Biosystems) were obtained from a previous study (19), in which they were divided into the same classes of expression as abovementioned for IDO2.

Moreover, the localization of the label of IDO2 in the peritumoral lung tissue was noted, according to histomorphological parameters to identify the various cellular types present.

### Statistical Analysis

Categorical variables were presented as frequencies with row and column percentages. Patients were divided into a young and an elderly group, according to the cut-off age (68 years, corresponding to patients' median age) for analysis. Categorical variables were compared between the groups (IDO2 low or IDO2 high) using Chi-square test or Fisher's exact test as appropriate. Odds Ratio (OR) was estimated when association was statistically significant.

Other causes of death were regarded as competing risk events in the patients' end-point. The cumulative incidence function (CIF) was compared between groups using Gray's method and was shown on a plot (20). Analysis of disease free survival (DFS) and overall survival (OS) were evaluated using a Fine and Gray model (competing risks regression in Supplementary Material 1) (21).

Continuous variables were categorized and the proportional hazards assumption of categorical variables was verified using a log-minus-log plot.

A *p*-value (*p*) < 0.05 was considered as statistically significant.

Statistical analyses were performed by STATA 15.1 (StataCorpLP, Collage Station TX, USA) (22).

## Results

### Patients Series

Data about patients' series were shown in Table 1.

One hundred and ninety-one patients were eligible for the study. Patients were all Caucasian, the median age was 68 years (range 38–84), with a median follow-up period of 50 months (range 1–107 months). One hundred and thirty-seven (72%) patients were males; 175 (91%) were either current smokers or former smokers. Regarding the pathological staging classification, 101 cases (53%) belonged to stage I, whereas 90 (47%) patients were in stage II-III. Fifty-six (29%), 16 (8%) and 1 (0.5%) patients relapsed after surgery, presenting 1, 2, or 3 localizations, respectively. Moreover, 12 patients presented nodal metastasis, 10 of which with one or more that were synchronous and hematogenous. Sixty-four (34%) died from NSCLC (Table 1).

### Pathological Findings

Data about pathological findings were summarized in Table 1.

Regarding histological characterization, the series was composed of 122 (64%) adenocarcinomas and 69 (36%) squamous cell carcinomas.

The most frequent predominant pattern of adenocarcinomas was the acinar (78; 64%).

Just over half of the adenocarcinomas (74; 61%) belonged to stage I, whereas the majority of squamous cell carcinomas (42; 61%) were in stage II-III.

### IDO2 Immunohistochemical Analysis

Concerning IDO2 evaluation, the majority of the tumors (160 cases, 84%) belonged to the high expression group of this molecule, both among adenocarcinomas (112; 92%) and among squamous cell carcinomas (48; 70%, Table 1).

**Figure 1:**
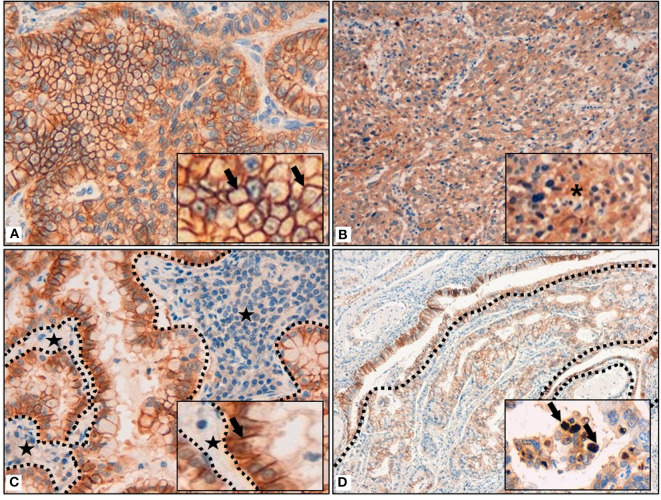
**(A)** Membrane reinforcement of IDO2, as black arrows shown in the inset. (**B)** Cytoplasmic expression of IDO2, as indicated in the inset by an asterisk. **(C)** IDO2 staining reinforcement at tumor-stroma interface. Dotted lines circumscribe the stroma and the black stars highlight it; IDO2 staining reinforcement is shown by the black arrow in the inset. **(D)** IDO2 bronchial epithelium staining (top left of the longer dotted line and circumscribed by the shorter dotted lines) and membranous tumoral staining (bottom right of the longer dotted line). Arrows in the inset highlight the nuclear staining of IDO2. Original magnification 400 × **(A,C)**, 200 × **(B)**, 100 × **(D)**; insets: 600 × **(A,C)**, 400 × **(B,D)**.

Most of the tumors (158; 83%) presented a membrane reinforcement of the stain (Figure 1A), with only 19 (12%) of those cases presenting a focal IDO2 labeling. In addition, 17 (11%) cases presented simultaneous cytoplasmic stains (Figure 1B) and, among these, only one had diffuse IDO2 expression. Eighty (51%) of the cases with only membranous immunostaining (which were 77–96% adenocarcinomas and 3–4% squamous cell carcinomas) presented IDO2 expression on the basolateral side of the tumor cellular membrane, with a reinforcement of the stain at the interface between tumor and stromal tissue and without an apical immunolabel (Figure 1C). Similarly, the immunostains presented a reinforcement at the interface between the tumor nest and healthy lung parenchima in 4 (6%) squamous cell carcinomas. Twenty cases (10%) also presented a nuclear pattern of staining (Figure 1D), most of which were in adenocarcinomas (19; 95%) seemed to highlight the nucleoli of the cells.

As for the peritumoral lung tissues, there was a constant IDO2 expression in bronchial epithelial cells, localized in their cytoplasm, with membrane reinforcement (Figure 1D); due to this aspect, we used this expression as an internal control for the labeling. We also found IDO2 in subepithelial bronchial glands with a diffuse pattern of staining.

In the lung parenchyma, IDO2 marked reactive pneumocytes close to tumor tissue and also intralveolar macrophages. In both cases there was a granular intracytoplasmatic staining.

### Clinical-Pathological Associations

The IDO2 associations with clinical-pathological parameters were reported in Table 1.

IDO2 showed a high expression when associated with a specific NSCLC histotype: in fact, in our series its high expression was found especially in adenocarcinomas (*p* < 0.001; OR = 4.9).

There were no correlations between IDO2 expression and the other clinical-pathological parameters examined, although there was almost a statistically significant association (*p* = 0.068) with patients who died from NSCLC: 91% presented a high IDO2 expression.

### Microenvironmental Associations

Data about associations between IDO2 and microenvironment molecules were shown in Table 1.

Interestingly, a high IDO2 expression correlated with high PD-L1 among the squamous cell carcinomas group (*p* = 0.012; OR = 6.2). On the other hand, among the adenocarcinomas group it was seen that the higher the expression of IDO2, the lower the expression of PD-L1 (*p* = 0.035; OR = 4.0).

There was no association between IDO1 and IDO2 expression, both in the adenocarcinomas and the squamous cell carcinomas groups.

It is worthy of note that among the adenocarcinoma subgroup, high IDO2 expression was associated with an intratumoral or mixed localization of the TILs (94 and 91% of the cases, respectively), in a statistically significant manner (*p* < 0.001; OR = 11.4). On the other hand, there was no association with IDO2 expression and TIL density, in either of the histotype groups.

### Survival Analysis

The results concerning the survival analysis were displayed in Tables 2, 3.

**Table 2:** Fine and Gray model on overall survival (OS).

**Parameter**	**Univariate analysis**			**Multivariate analysis**		
	***SHR^***a***^***	***p-value***	***95% CI^***b***^***	***SHR^***a***^***	***p-value***	***95% CI^***b***^***
**IDO2**						
Low	ref	–	–	ref	–	–
High	2.64	**0.028**	(1.11–6.31)	2.94	**0.011**	(1.28–6.77)
**IDO1**						
Low	ref	–	–	ref	–	–
High	1.71	**0.041**	(1.02–2.85)	1.64	**0.041**	(1.12–2.76)
**SEX**						
Female	ref	–	–	ref	–	–
Male	2.03	**0.029**	(1.08–3.85)	2.22	**0.019**	(1.14–4.31)
**STAGE**						
I	ref	–	–	ref	–	–
II–III	1.88	**0.011**	(1.16–3.07)	1.99	**0.005**	(1.23–3.24)
**HISTOTYPE**						
Adc^c^	1.41	0.189	(0.84–2.37)	–		
Sqcc^d^	ref	–	–			
**AGE**						
<68 years	ref	–	–	–		
≥68 years	1.04	0.887	(0.64–1.69)			
**SMOKING**						
Current smoker	1.86	0.219	(0.69–5.04)	–		
Former smoker	1.60	0.350	(0.60–4.29)			
Never smoker	ref	–	–			
**PD–L1**						
Low	1.04	0.904	(0.57–1.87)	–		
High	ref	–	–			
**TILs DENSITY**						
Low	ref	–	–	–		
High	1.05	0.841	(0.65–1.71)			
**TILs LOCALIZATION**						
Intratumoral	1.04	0.972	(0.12–9.19)	–		
Peritumoral	1.45	0.752	(0.14–10.81)			
Mixed	1.24	0.848	(0.14–10.81)			
Absent	ref	–	–			

a *SHR: Subdistribution Hazard Ratio*.

b *CI: Confidence Interval*.

c *Adc: adenocarcinoma*.

d *Sqcc: squamous cell carcinoma*.

**Table 3:** Fine and Gray model on disease free survival (DFS).

**Parameter**	**Univariate analysis**			**Multivariate analysis**		
	***SHR^***a***^***	***p-value***	***95% CI^***b***^***	***SHR^***a***^***	***p-value***	***95% CI^***b***^***
**IDO2**						
Low	ref	–	–	–		
High	1.03	0.937	(0.55–1.91)			
**IDO1**						
Low	ref	–	–	–		
High	1.44	0.133	(0.89–2.31)			
**SEX**						
Female	ref	–	–	–		
Male	1.22	0.461	(0.72–2.05)			
**STAGE**						
I	ref	–	–	ref	–	–
II–III	1.60	**0.044**	(1.01–2.54)	1.92	**0.006**	(1.20–3.09)
**HISTOTYPE**						
Adc^c^	1.96	**0.012**	(1.16–3.32)	2.31	**0.002**	(1.35–3.96)
Sqcc^d^	ref	–	–	ref	–	–
**AGE**						
<68 years	ref	–	–	–		
≥68 years	0.72	0.163	(0.45–1.14)			
**SMOKING**						
Current smoker	1.91	0.173	(0.75–4.87)	–		
Former smoker	1.23	0.663	(0.47–3.17)			
Never smoker	ref	–	–			
**PD–L1**						
Low	1.06	0.834	(0.60–1.88)	–		
High	ref	–	–			
**TILs DENSITY**						
Low	ref	–	–	–		
High	1.00	0.986	(0.63–1.58)			
**TILs LOCALIZATION**						
Intratumoral	1.80	0.563	(0.25–13.15)	–		
Peritumoral	2.54	0.380	(0.31–20.34)			
Mixed	1.50	0.691	(0.20–11.01)			
Absent	ref	–	–			

a *SHR: Subdistribution Hazard Ratio*.

b *CI: Confidence Interval*.

c *Adc: adenocarcinoma*.

d *Sqcc: squamous cell carcinoma*.

**Figure 2:**
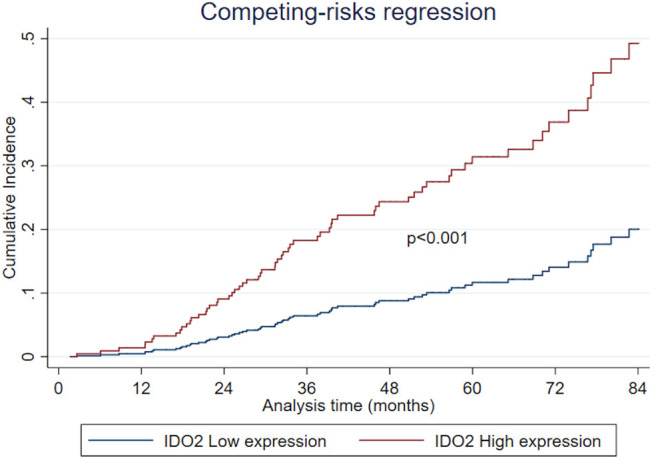
The cumulative incidence of IDO2 expression after competing-risks regression.

Regarding the univariate analysis, the most relevant statistically significant associations were between the increased probability of death from NSCLC and high expression of both IDO2 (SHR 2.64, 95% Confidence Interval—CI−1.11–6.31, *p* = 0.028) and IDO1 (SHR 1.71, 95% CI 1.02–2.85, *p* = 0.041); these relationships persisted also in the multivariate analysis, which highlighted a greater probability of death for the patients with a tumor high expression of either IDO2 or IDO1 than the NSCLC with a low expression level of these molecules (SHR 2.94, 95% CI 1.28–6.77, *p* = 0.011 and SHR 1.64, 95% CI 1.12–2.76, *p* = 0.041, respectively; Table 2). In addition, regarding patients with a high tumor expression of IDO2, the probability of death within 36 months was roughly 18% compared to almost 7% for the group with a low expression of IDO2 (*p* < 0.001). This difference increased within 60 months (28 vs. 12%, respectively, *p* < 0.001; Figure 2).

Similarly, both in the univariate and in the multivariate analysis, either being a male patient or presenting a stage II-III of disease increased the probability of death from NSCLC (Table 2).

The histotype, age of the patient, smoking habits, expression of PD-L1, TILs density and TILs localization had no statistically significant correlations with the probability of death from NSCLC (Table 2).

Belonging to either the stage II-III group or the adenocarcinoma group increased the risk of recurrence in the present NSCLC series, both regarding the univariate (SHR 1.60 and 1.96; 95% CI 1.01–2.54 and 1.16–3.32; *p* = 0.044 and 0.012, respectively) and the multivariate analysis (SHR 1.92 and 2.31; 95% CI 1.20–3.09 and 1.35–3.96; *p*= 0.006 and 0.002, respectively), as reported in Table 3.

IDO2 and the other parameters considered showed no association with the DFS.

## Discussion

Little is known about the role of indoleamine 2,3-dioxygenase 2 (IDO2) and its implications both in normal lung tissue and in NSCLC. In this scenario, we examined IDO2 immunolabeling in 191 resected NSCLC cases, in order to better understand its expression in this cancer type and to determine its correlations with clinical-pathological parameters, other immunomodulatory molecules and patients' prognosis.

Unlike IDO1, the real IDO2 cellular function is poorly understood even today, in both normal and tumor cells. As a matter of fact, it seems to have no—or just low—enzymatic activity on tryptophan, so some other mechanisms could be involved to explain its putative role in the tumoral immunoescape (6, 11, 13, 14, 23–26).

Previous studies demonstrated a constitutive expression of only IDO2 mRNAs in human liver, small intestine, spleen, brain, thyroid, placenta, thymus, lung, kidney, colon, endometrium and testis, with a full length and functional transcript highlighted only for the placenta and brain (6, 27–29). Surprisingly we found, as an incidental observation, an almost constant immunohistochemical IDO2 staining of both the bronchial epithelium and peribronchial sub-epithelial glands. The continuous exposure of the airways, particularly the upper ones, to external stimuli could explain the induction of a putatively tolerogenic IDO2 in the abovementioned tissues, as happens in antigen presenting cells (APCs) or in B cells during either inflammatory or reactive states (25–28).

Furthermore, the IDO2 labeling of both reactive pneumocytes and alveolar macrophages, observed in the current study, seems to confirm the existence of an adjunctive mechanism triggering IDO2 expression under specific microenvironmental conditions, such as stress. Nevertheless, if the immunohistochemical expression corresponds to a functionally active IDO2 protein (6, 16, 29) in human lung tissues it would need further studies.

Another interesting and incidental finding is that 10% of NSCLCs presented a nuclear pattern of IDO2 staining in tumor cells. This was already observed in murine series regarding hepatocytes (13). Moreover, in a previous study (19) we reported a nuclear labeling for IDO1 in NSCLC. However, it is generally not known how these two molecules would act at nuclear level, but the observation that some tumors present a nuclear localization of both IDO1 and IDO2 may suggest a signal-transducing function (13, 30), something already noted about IDO1 (31).

Furthermore, the consistent percentage (83%) of NSCLC in our series with an intense membranous IDO2 immunolabeling might open the way to further studies about its correlation with adhesion molecules, such as those from the cadherin family. It is known how the latter are involved in epithelial-mesenchymal transition in an Aryl hydrocarbon Receptor (AhR)-kynurenine dependent manner (32, 33), and that the kynurenines are in turn the product of IDO1 enzymatic activity. On the other hand, it is also known that IDO2 is not expressed as a functional tryptophan-degrading enzyme (6), at least not in human cancer cells lines (14). As an alternative, other authors (29) have correlated the IDO2-dependent/tryptophan-independent activation of an inhibitory isoform of immunoregulatory transcription factor NF-IL6 (LIP) to a potential IDO2 role in metastatization. Consequently, a difference between IDO1 and IDO2 activity may really exist. These findings could support either a direct role of IDO2 in cellular adhesion (13) or an indirect role in modulating other adhesion molecules, promoting tumor invasiveness and transition toward a mesenchymal and more aggressive phenotype. Regarding the correlation between this IDO2 localization and the patients' prognosis, we did not find any statistically significant results. Furthermore, the majority of the NSCLCs with such an immunolabeling pattern were, interestingly, adenocarcinomas. Moreover, this histotype often (64%) has a basolateral staining of the tumor cells, possibly related to the presence of intracytoplasmic mucus, which is a characteristic of the adenocarcinoma, in particular of the most differentiated ones. In addition, a high IDO2 level was more frequently present in adenocarcinomas than in the squamous cell carcinoma subgroup, a finding that corroborates the strict relationship between this molecule and the specific microenvironment of this NSCLC histotype. Despite the fact that IDO2 action in adenocarcinomas and in adenocarcinoma patterns may differ from that of IDO1 (6, 11, 14, 17–19, 24–26), we could speculate about the existence of other immunosuppressive mechanisms induced by IDO2 in this NSCLC subgroup. An alternative splicing of IDO2 (6, 13) could explain the different localizations found and suggest the occurrence of a distinctive splicing induction under certain conditions, such as inflammatory states, or according to particular tumor histotype—adenocarcinomas—as above reported. Some authors suggest that IDO2 activation is related to specific microenvironmental conditions (13, 26, 34) in specific cell types (11, 25), such as the neoplastic ones in our study, which is consistent with its possible immunomodulatory role in either stress conditions or disease response (13, 25).

Interestingly, we found a high co-expression of both PD-L1 and IDO2 in the squamous cell carcinomas subgroup, further evidence that IDO2 expression occurs in cells displaying tolerance markers. Based on this finding, a dual combination NSCLC therapy, such as inhibitors of both PD-1/PD-L1 immune checkpoints and IDO2, might be hypothesized. Currently, the combination of immunocheckpoint inhibitors and IDO1 hinderers has already been tested in ongoing clinical trials, with encouraging results in NSCLC patients (35, 36). This approach could be easily transposed into further researches targeting combination therapies including IDO2 inhibitors.

Moreover, the fact that high IDO2 expression is associated, among adenocarcinomas, with intratumoral and mixed localization of TILs could suggest a possible role for IDO2 as an immunomodulatory molecule. As a matter of fact, it is partly already known how IDO2 could be involved in B cell-mediated autoimmunity (23, 37) and may also influence Treg activation (37). Although in some murine models IDO2 has been associated with a potential pro-inflammatory role, particularly in autoimmune diseases (38), other authors showed that IDO2 contributions to inflammation, both in the context of cancer and autoimmune disorders, remains to be elucidated (38, 39). Moreover, Metz et al. (26) demonstrated an immune modulation role of IDO2, and distinguished its non-redundant contributions to inflammation. Consequently, the increased IDO2 expression in NSCLC could likely occur when the tumor cells are closely in contact with the inflammatory infiltrate, and could be interpreted as a tumor attempt to evade the immune system attack (40–43).

Furthermore, there is a strict correlation, never described before, between high IDO2 expression and a worse NSCLC outcome. Moreover, from the long follow-up period we highlighted an increasing difference in the probability of death between the patients belonging to the group with a high tumor expression of IDO2 and those belonging to the low expression group (28% compared to near 12% within 60 months). This finding could suggest a delayed role for IDO2 in both NSCLC progression and aggressiveness, which deserves further investigation.

Although many efforts have been made in order to identify prognostic molecules for NSCLC, nowadays the results are still conflicting (17, 18, 44–50). In this regard, the lack of a statistically significant correlation between DFS and the high tumor expression of both IDO2 and IDO1 could appear to be a confounding result, in particular when compared to the OS analysis of the current series. Despite the fact that some studies have found an association between IDO1 expression and disease progression (7, 51–55), some other authors have claimed that there was no impact on survival, regarding both DFS and OS (17, 56–59). Nevertheless, a focus on the highly versatile nature of IDO1 might explain this contradiction, because IDO1 has not only an enzymatic activity, but also a signaling function (31). Therefore, IDO1 is reported to be related to both immunoescape and inflammatory responses, strictly depending on the surrounding microenvironment (31, 60), and its expression could relate to a wide spectrum of patients' outcomes (61). Regarding IDO2, we could assume a similar role, resulting both in the induction of and in the resistance to the host's immune system (13, 23, 26, 34, 37, 38, 60); consequently, IDO2 could be implicated either in delaying or promoting tumor aggressiveness, based on the highly fluctuating interactions with all of the other activated molecules of the tumor microenvironment (11, 13, 25, 26, 34). However, additional studies are needed to demonstrate this, since IDO1 and IDO2 seem to be functionally different (12, 13, 31, 60) and the biological relevance of IDO2 is not fully understood yet (60, 62).

On the other hand, encouraging evidence about the prognostic role of both IDO1 and PD-L1 in NSCLC has been found (19), as confirmed in the current study by the correlation between the IDO1 overexpression and the high probability of death from cancer. At the moment, we could suggest the immunohistochemical assessment of IDO2 together with the abovementioned molecules, in order to better stratify the risk of patients with NSCLC, assuming that more than one biomarker influences, in an independent manner, the outcome of these tumors.

The present study supports the idea that there is the need to combine multiple biomarker assays, due to the multifactorial and complex nature of cancer-immune interactions (63, 64).

To the best of our knowledge, this is the first study about IDO2 immunohistochemical expression in NSCLC. The close relationships found between IDO2 and other molecules in the NSCLC microenvironment, together with its potential prognostic implications, could open the way for the assessment of possible combined therapeutic strategies with IDO2 selective inhibitors, both by figuring new mechanisms out and by exploring new pharmacological tools for NSCLC. The objectives are both to overcome the existing drug resistances and to increase the number of patients who could benefit from immunotherapy in this cancer type. Due to the so far limited knowledge of IDO2 expression and cellular functions, further studies at a molecular level are required to make this promising molecule become a new biomarker for NSCLC.

## Data Availability Statement

The datasets analyzed in this article are not publicly available in order to respect the confidentiality and protection of patients' data, in compliance with the processing of data covered and protected by the Italian Privacy Law and by the GDPR (General Data Protection Regulation, EU regulation no. 2016/679). However, requests for access to a properly anonymized dataset of the present article could be directed to MM, mandaranomartina@gmail.com.

## Ethics Statement

The studies involving human participants were reviewed and approved by Comitato Etico delle Aziende Sanitarie della Regione Umbria (CEAS Umbria), Regione Umbria Palazzo Broletto Floor 3, Via Mario Angeloni 61, 06124 Perugia, Italy. The patients/participants provided their written informed consent to participate in this study.

## Author Contributions

MM, GB, MB, CV, and AS conceived and designed the study. JV, LC, and FP provided the resected surgical specimens. IF and GF performed both the histological slides and the immunohistochemical stains. MM, GB, and AS performed the histopathological analysis. MM, GB, MB, CV, GMo, JV, RC, VL, GMe, and FR filed and analyzed data. AG, CL, and FS provided the follow up data and performed the statistical analysis. MM, GB, MB, and CV wrote the paper. All the authors edited and contributed to manuscript revision, giving the final approval for publication.

## Conflict of Interest

The authors declare that the research was conducted in the absence of any commercial or financial relationships that could be construed as a potential conflict of interest.
