# Supplementary material for: Indoleamine 2,3-Dioxygenase 2 Immunohistochemical Expression in Resected Human Non-small Cell Lung Cancer: A Potential New Prognostic Tool
Source: Front Immunol. 2020 May 27;11:839. doi: 10.3389/fimmu.2020.00839 (PMC7267213; doi:10.3389/fimmu.2020.00839)
Supplement: Supplementary file 1 [file Data_Sheet_1.docx]

For analyzing competing risks data, standard survival analysis method, e.g. Cox Proportional Hazard (PH) model, has been commonly used. There exists a number of limitations in using this model. As remedy, Fine and Gray proposed the cumulative incidence function(CIF) based PH model to analyze survival data arising from a competing risk setup (Coviello V, Boggess M. Cumulative incidence estimation in the presence of competing risks. The Stata J. 2004;4(2):103–12) In the competing risks setup, under each cause for the occurrence of an event of interest, a hazard function in the presence of covariates is considered. The number of failures from the causes other than the cause of interest reduces the actual number of failures from the cause of interest. Therefore, the estimated probability observed for the cause of interest may be underestimated (Gooley TA, Leisenring W, Crowley J, Storer BE. Estimation of failure probabilities in the presence of competing risks: new representations of old estimators. Stat Med. 1999;18:695–706.). To take care of this, Fine and Gray developed a survival regression model using the CIF and sub-distribution hazard functions. The parameters involved in the model are estimated by incorporating weights in the partial likelihood function. Under this model, for a covariate xr, the sub-distribution hazard ratio (SHR) for the cause j (j=1,⋯,p) is given by exp(βjr) keeping all other covariates at a fixed level, where βjr is the regression coefficient.
